# Evaluating the Vulnerability of Several Geodetic GNSS Receivers under Chirp Signal L1/E1 Jamming

**DOI:** 10.3390/s20030814

**Published:** 2020-02-03

**Authors:** Matej Bažec, Franc Dimc, Polona Pavlovčič-Prešeren

**Affiliations:** 1 Faculty of Maritime studies and Transport, University of Ljubljana, Cesta pomorščakov 4, 6320 Portorož, Slovenia; matej.bazec@fpp.uni-lj.si (M.B.); franc.dimc@fpp.uni-lj.si (F.D.); 2 Faculty of Civil and Geodetic Engineering, University of Ljubljana, Jamova cesta 2, 1000 Ljubljana, Slovenia

**Keywords:** GNSS jammer, geodetic GNSS receivers, signal loss, carrier-to-noise-ratio

## Abstract

Understanding the factors that might intentionally influence the reception of global navigation satellite system (GNSS) signals can be a challenging topic today. The focus of this research is to evaluate the vulnerability of geodetic GNSS receivers under the use of a low-cost L1 GPS band and E1 Galileo frequency band (L1/E1) frequency jammer. A suitable area for testing was established in Slovenia. Nine receivers from different manufacturers were under consideration in this study. While positioning, intentional 3-minute jammings were performed by a jammer that was located statically at different distances from receivers. Furthermore, kinematic disturbances were performed using a jammer placed in a vehicle that passed the testing area at various speeds. An analysis of different scenarios indicated that despite the use of an L1/E1 jammer, the GLONASS (Russian: Globalnaya Navigatsionnaya Sputnikovaya Sistema) and Galileo signals were also affected, either due to the increased carrier-to-noise-ratio (C/N_0_) or, in the worst cases, by a loss-of-signal. A jammer could substantially affect the position, either with a lack of any practical solution or even with a wrong position. Maximal errors in the carrier-phase positions, which should be considered a concern for geodesy, differed by a few metres from the exact solution. The factor that completely disabled the signal reception was the proximity of a jammer, regardless of its static or kinematic mode.

## 1. Introduction

In today’s world, the global navigation satellite system (GNSS) presents an indispensable source of positioning, navigation and timing information for military and civilian users, as well as for several other sectors. The continuous availability of the GNSS means that a wide variety of sectors and industries rely on it, including transportation, emergency services, industry, communication, finance, government and various global/regional/national infrastructures that use geodetic features. The growing dependency on the GNSS leads to greater demands for the availability and reliability of acquisition of the GNSS signal. From the very outset of the GNSS, there has been an awareness of the most serious natural sources of disturbances that can affect the positioning of the GNSS, for which several effective processing strategies and filters have emerged or are still under development. Unintentional disturbances, multipath and interference are considered major sources of errors that can be very harmful to GNSS signals and further positioning. At first, it was assumed that the problem of intentional malicious threats was not a major concern for civil users. Jammers served naval information warfare systems command (NAVWAR) strategies and were mostly under the military domain. Today, however, this problem has changed drastically due to the increasing dependency on the GNSS and the availability of low-cost jammers, such as personal privacy devices (PPDs). Several authors [1,2,3] have warned that the dependency on the GNSS and the vulnerability of the navigation process due to jammers can cause severe problems. They also stress that the impact of jamming is often not clear for users. Since jammers can cause great damage, it is important to recognise their influence on the operation of different instruments, especially those that play a crucial role in establishing the fundamental infrastructure connected to assisted navigation or positioning. 

In today’s geodesy, the GNSS presents a primary source for the establishment and maintenance of fundamental geodetic infrastructure, which is based on Continuously Operating Reference Stations (CORS). The establishment of CORS networks started in the mid-1990s. During the last two decades, the demand for the optimal continuous functioning of CORS has increased significantly. The optimal functioning of CORS networks is one of the major concerns since (a) modern geodetic coordinate systems rely on a time series of the determination of coordinates [4], and (b) they must provide users with corrections to improve their precision for every specific moment of a day. CORS also support three-dimensional positioning in autonomous driving using high accuracy localisation based on techniques such as differential real-time kinematic (RTK) or precise point positioning (PPP) [5,6,7]. Such techniques require good signal reception, both at base stations and on users’ receivers, to achieve positioning accuracy within a few centimetres.

Since permanently installed GNSS receivers at stations have been able to provide continuous monitoring of changes in position, they have also started to serve geophysical and geodynamics applications. Several GNSS processing strategies have been introduced to minimise positioning errors for estimations of the various impacts of the GNSS, for example, zenith tropospheric and ionospheric delay, which can be used beyond positioning applications. Such products are useful in meteorology for short-term weather prediction [8,9], space weather monitoring [10,11,12] and other geophysical applications, including seismic applications [13]. 

To meet the basic required needs of geodesy and the geosciences, the CORS infrastructure consists of stations equipped with GNSS receivers, which collect data from at least one, though usually multiple, navigations systems (GPS (Global Positioning System), GLONASS (Russian: Globalnaya Navigatsionnaya Sputnikovaya Sistema), Galileo, BeiDou (Chinese: Běidǒu Wèixīng Dǎoháng Xìtǒng)/COMPASS, SBAS (Satellite-Based Augmentation System), QZSSS (Quasi-Zenith Satellite System), IRNSS (Indian Regional Navigational Satellite System)). The widespread use of real-time positioning requires additional communication links between a reference and rover receiver. This can result in the GNSS infrastructure being vulnerable to natural impacts (extreme Sun events), multipath and interference caused by unintentional or intentional degradation of the signal, as well as on the communications links. This paper, however, focuses on the impact of GNSS jamming, while real-time communication problems are not considered.

Contrary to natural perturbations, the degradation or replacement of the GNSS signal should be the greatest concern. Non-intentional degradation can happen in areas within radars or amateur radios that share the same GNSS bands. Their static position makes them identifiable by interference monitoring systems [14,15]. Deliberate errors come from malicious threats using GNSS jammers or spoofers, which usually do not hold static, so they cannot be easily traced. Finding a solution for intentional disturbances is one of the major concerns in the upcoming era of growing autonomous navigation systems (ANS). Jammers, as a kind of signal blocker for the GNSS, are radio frequency transmitters, which interfere or, in the worst cases, block signal reception. Since they present a serious risk to public safety, several national laws prohibit their use. On the contrary, however, marketing and selling such jammers over the internet is not prohibited nor regulated. The fact that people with some electro-technical skills could build a GNSS jammer by themselves is of great concern. 

It is a known fact that GNSS geodetic receivers from different manufacturers follow specific internal filtering processes that can mitigate effects, for example multipath [16]. While studying the effects of jamming, different approaches in the detection and mitigation of the GNSS multipath with a combination of signal-to-noise-ratio (SNR) measurements can be used as the reference [17]. Manufacturers of receivers have recently been putting huge efforts into research to overcome the problems of the intentional degradation of signals. Although they have their own specific anti-jamming solutions, they all include antenna-based and receiver-based techniques into their solutions. They produce receivers with signal-degradation mitigations or even equip them with additional sensors, for example, inertial measurement units (IMUs). Chips within instruments incorporate spectrum analysers, surface acoustic wave (SAW) and more sophisticated thin-film bulk acoustic resonators (FBAR). Adaptive notch filters are an effective solution used to mitigate jamming and extend the capabilities of GNSS operations. These filters are capable of tracking frequency variations of a jamming signal, pulse blanking or adaptive beamforming based on the multi-antenna solution [18,19]. The use of controlled reception pattern antennas (CRPA) is beneficial since they provide significant anti-jamming protection [20]. Despite significant improvements, local interference from nearby receivers remains, meaning there are severe issues to be resolved in the future [21]. 

As a result of previous studies carried out at the University of Ljubljana and those from the literature, the main objective of this research is to investigate the impact of L1/E1 carrier frequency (1575.42 MHz) jammers on geodetic receivers from different manufacturers and generations. In order to achieve this, receivers were tested simultaneously in the field in real jamming scenarios. Among the many techniques for analysing the impact of a jammer, the authors limited themselves to changes in the observed carrier-to-noise density (C/N_0_) values of the received signals and differences in coordinates acquired from GNSS processing while jamming. The reason was that quality positioning is one of the major concerns in geodesy.

### 1.1. Previous Research

In order to protect GNSS users, several studies have addressed the problem of the detection and localisation of interference. Experiments testing the localisation of jammers have been performed indoors in laboratories [22,23,24], and in some cases, also in real open-field conditions. The latter have been performed in maritime conditions in the United Kingdom [25] and in the northern part of Norway [26]; in-car jammers have been tested in Germany [27], South Africa [28] and Slovenia [29]; while studies of jamming in aviation have been performed in Switzerland [30]. Since the prohibition of the use of jammers, civilian research studies in real scenarios are now limited, restricted or even prohibited in most countries. However, several military exercises, which can also impact in-car or network receivers, have been or are due to be performed in 2020 in the United Kingdom [31]. Several algorithms have been introduced for in-car GNSS jammer localisations [32] (see, for example, Cheng et al. [33]). The possible effect of terrain on the jamming signal propagation was tested in van Niekerk and Combrinck [28]. The main finding of the study was that terrain charasteristics can mitigate the effects of a ground-based jammer. A localisation method in urban canyons for in-car jammers, which work on monitoring networks and follow the principle of pattern recognition, is described in Lyu et al. [34]. In Kuusniemi and Airos [24], the effect of single-frequency jammers on consumer grade receivers was analysed. The goal was to estimate the availability and positioning quality of the solution. In the severe jamming scenario, an enormous amount (75%) of availability of positioning was enabled, which led to the quality of positioning of the remaining amount being severely affected.

In an extensive study of the performance of GPS in maritime environments under jamming conditions in the North Sea in 2014 [26], the Leica GS10 geodetic receiver and the Leica AS10 antenna were included. An important finding of this study was that by using an L1/E1 jammer, GLONASS signals remained resistant to jamming. Based on the authors’ knowledge, no experiments have yet been conducted on the jamming of several geodetic GNSS receivers of different manufacturers, as well as generations that operate simultaneously in the same location. To the contrary, several studies have analysed the effects of various jammers, for example, the use of a single-frequency or dual-frequency jammers [24,35].

### 1.2. Paper Focus and Outline

Within the context of the research field, the driving motivation for this research was to conduct some further real condition jamming experiments and analyse the results in order to gain a better understanding of the performance of geodetic GNSS receivers when faced with an intentional degradation of signals. The focus of this paper is based on an estimation of the vulnerability of geodetic GNSS receivers under various L1/E1 jamming scenarios. More specifically, the research focused on two different issues: (a) the impact of static jamming where the distances between the jammer and receivers varied, and (b) the impact of short-term kinematic intentional disturbances, both on signal quality and positional (horizontal and vertical) accuracy. 

The outline of the paper is as follows. The second section (Section 2) discusses the theory. Basic facts are presented on the GNSS positioning and ambiguity fixing algorithms, followed by an introduction to the carrier-to-noise-ratio. This section also covers the localisation of moving jammers and the dispersion of receiver coordinates while jamming. The next section (Section 3) describes the setup and measurement campaign and the receivers and jammer used in the experiment, followed by a description of the experiments (Section 4). Section 5 contains data analysis, results and discussion, followed by a summary of the main findings and a description of further ideas for testing (Section 6).

## 2. GNSS Use in Geodesy and Jamming

Several methods can be used to achieve high accuracy positioning in geodesy. Since static positioning can be time-consuming, kinematic positioning, especially the RTK method, is the most popular method used in most everyday geodetic applications. Its greatest advantage is the ability to acquire quality positioning in real time. Moreover, it enables immediate determination of coordinates in the national coordinate system, which is indispensable in the land cadastre. The typical nominal accuracy of RTK is 1 cm ± 2 ppm in a horizontal plane and 2 cm ± 2 ppm vertically or better. The basic concept is based on relative positioning to reduce and remove errors common to a base station and rover pair. This implies the fact that difficulties in positioning due to the presence of a jammer can occur in two situations: (a) at a base receiver or (b) at the location of a rover.

GNSS relative positioning follows the scenario in which a priori coordinates are defined from absolute code positioning first. Single-point positioning, which is also known as a navigation solution, is based on a single receiver measuring satellites simultaneously. In this, the pseudoranges $P_{A}^{i}$ from code measurement between the satellite i and the receiver A are used [36]:

(1) $$P_{A}^{i}=Z_{A}^{i}+\Delta \rho_{A}^{i}=r_{A}^{i}+c\left(\delta t_{A}+\delta t^{i}\right)+O^{i}+T_{A}^{i}+I_{A}^{i}+M_{A}^{i}+\delta P_{A}^{i}+\cdots$$

In the above equation $Z_{A}^{i}$ is an unambiguous time measurement from the navigation message and $\Delta \rho_{A}^{i}$ stands for the code measurement, which is unambiguous beyond the code length. The right-hand side of the Equation (1) shows that code measurements, apart from the distance to the satellite $r_{A}^{i}$, contain the $\delta t_{A}$ and $\delta t^{i}$ receiver and satellite clock errors, respectively, $O^{i}$, $T_{A}^{i}$, $I_{A}^{i}$ and $M_{A}^{i}$ are the orbital, tropospheric, ionospheric and multipath errors, respectively, and $\delta P_{A}^{i}$ is the code noise. Measurements from at least four satellites must be available to compute the unknown position and time.

Besides the code, carrier-phase (or shorter phase) measurements $\Phi_{A}^{i}$ can be used to obtain the distance between the satellite i and the receiver A [36]:

(2) $$\Phi_{A}^{i}=\lambda \cdot N_{A}^{i\left(0\right)}+\Delta \varphi_{A}^{i}=r_{A}^{i}+c\left(\delta t_{A}+\delta t^{i}\right)+O^{i}+T_{A}^{i}-I_{A}^{i}+M_{A}^{i}+{\delta \Phi }_{A}^{i}+\cdots$$

In Equation (2), the additional unknown $N_{A}^{i\left(0\right)}$ of the carrier-phase ambiguity number exists. As before,$\;\delta t_{A}$ and $\delta t^{i}$ stand for the receiver and satellite clock errors, respectively; $O^{i}$, $T_{A}^{i}$, $I_{A}^{i}$ and $M_{A}^{i}$ are the orbital, tropospheric, ionospheric and multipath errors, respectively; and ${\delta \Phi }_{A}^{i}$ is the carrier-phase noise. The ionospheric term $I_{A}^{i}$ has opposite signs for the code and carrier phase, which is due to the fact that the ionosphere produces an advance in the carrier-phase measurements equal to the delay on the code measurements. 

A positioning technique from the carrier-phase measurements that removes or models GNSS system errors to provide a high level of position accuracy from a single receiver is known as precise point positioning (PPP) [5,6,7]. In geodesy, the method is used mostly in post-processing since the time necessary to move from a float to a fixed solution is extended and the convergence can take several tens of minutes. Currently, relative carrier-phase-based methods, especially RTK, are still the most used geodetic positioning techniques.

Relative GNSS carrier-phase positioning is based on the use of two receivers at two locations and on forming single, double and triple differences to reduce common errors for both (base and rover) receivers. The code and carrier-phase observations are acquired from the same (at least four) satellites *i* at two locations *A* and *B*, respectively [36]:

(3) $$\begin{array}{c} P_{A}^{i}=Z_{A}^{i}+\Delta \rho_{A}^{i}=r_{A}^{i}+c\left(\delta t_{A}+\delta t^{i}\right)+O^{i}+T_{A}^{i}+I_{A}^{i}+M_{A}^{i}+\delta P_{A}^{i}+\cdots ,\\ \Phi_{A}^{i}=\lambda \cdot N_{A}^{i\left(0\right)}+\Delta \varphi_{A}^{i}=r_{A}^{i}+c\left(\delta t_{A}+\delta t^{i}\right)+O^{i}+T_{A}^{i}-I_{A}^{i}+M_{A}^{i}+{\delta \Phi }_{A}^{i}+\cdots ,\\ P_{B}^{i}=Z_{B}^{i}+\Delta \rho_{B}^{i}=r_{B}^{i}+c\left(\delta t_{B}+\delta t^{i}\right)+O^{i}+T_{B}^{i}+I_{B}^{i}+M_{B}^{i}+\delta P_{B}^{i}+\cdots ,\\ \Phi_{B}^{i}=\lambda \cdot N_{B}^{i\left(0\right)}+\Delta \varphi_{B}^{i}=r_{B}^{i}+c\left(\delta t_{B}+\delta t^{i}\right)+O^{i}+T_{B}^{i}-I_{B}^{i}+M_{B}^{i}+{\delta \Phi }_{B}^{i}+\cdots , \end{array}$$

$P_{A}^{i}$ and $P_{B}^{i}$ are code and $\Phi_{A}^{i}$ and $\Phi_{B}^{i}$ are carrier-phase observations at locations *A* (base) and *B* (rover) from satellite *i*, expressed in metres; $\Delta \varphi_{A}^{i}$ and $\Delta \varphi_{B}^{i}$ are carrier-phase measurements that are unambiguous in the fixed number of wavelengths; and ambiguity numbers $N_{A}$ and $N_{B}$ have to be resolved using algorithms. The satellite clock error $\delta t^{i}$, as well as the orbital error $O^{i}$, are the same for the base station and rover receiver, while other errors are receiver or location dependent.

For the base receiver (at point *A*) for which coordinates are known, most of the effects in Equation (3) can be defined numerically. Estimated errors at the base can be used in the computation process of correcting ambiguity, provided that the location of the rover is close to that of the base receiver. However, some errors (mostly multipath, or in the worst-case, intentional degradation of the signal) remain, which can significantly affect the positioning. 

Methods that allow for the resolution of ambiguities in relative positioning are based on double differences because they do not contain changing clock errors. The goal of algorithms is to find ambiguity numbers, which minimise the residuals of double differences of carrier-phase observations (∇Δ), given as [36]:

(4) $$\sum_{i=1}^{n-1}\nabla \Delta v^{i}=\sum_{i=1}^{n-1}\nabla \Delta r^{i}-\nabla \Delta \Phi^{i}-\lambda \cdot \nabla \Delta N^{i}-\nabla \Delta {\delta \Phi }^{i}.$$

In Equation (4), Δ and ∇ present single and double difference operators, respectively. RTK algorithms must satisfy the condition$\;\nabla \Delta v^{i}\to 0$. Several algorithms can be used for ambiguity fixing. The least squares ambiguity decorrelation adjustment algorithm (LAMBDA) [37] and modified LAMBDA (MLAMBDA) algorithm [38] have been proven to theoretically be the most efficient integer ambiguity algorithms. LAMBDA reduces the computational complexity, thus allowing for significant development of the RTK method. 

The algorithms of RTK are based on the continuous tracked measurements to perform the initialisation of the carrier-phase ambiguities. Interruption of a signal at one of the receivers (either *A*, *B*, or both) requires a new initialisation process (ambiguity fixing). In the event of interruptions at a rover’s location, only the user is affected. Interruptions at the location of a base receiver, from which (usually) many users acquire observations for positioning, are far more severe. Therefore, CORS networks address issues, such as the high reliability of real-time distribution and quality control of the data they collect. The occurrence of the disruption of jammers should be another aspect to consider.

The problem for RTK users is that they often only produce coordinates without storing raw observations for additional observation quality checks. In cases of raw observations storage, a post-process quality check can be performed, which allows for a more detailed insight into the quality of the observations from which the final coordinates were obtained. For this purpose, determination of the signal-to-noise (SNR) or carrier-to-noise ratio (C/N_0_) and multipath effects, based on the Melbourne–Wübbena linear combination of code and carrier-phase observations, are the most effective estimates to describe the measurement conditions in the field. The first is related to unintentional disturbances, while the latter is related to both intentional and unintentional disturbances.

GNSS instruments for geodetic applications usually do not use inertial measurement units (IMUs) since their aim is the continuous reception of raw GNSS signals, which are used in further processing, in either RTK or post-processing. Reception of raw GNSS observations is essential since positioning is based on the determination of coordinates only from the GNSS, or, in some cases, through a combination of the GNSS and terrestrial geodetic measurements. Knowledge that disruptions can cause positioning problems, either due to the inability of signal reception or wrong ambiguity fixing, is essential. The awareness of mistaken positioning should be taken into consideration since the coordinates are used in further computation.

### 2.1. Signal-to-Noise-Ratio and Carrier-to-Noise Density Power Ratio

The signal-to-noise-ratio (SNR) is used to express the signal strength, which implies the signal quality. It can be estimated during the correlation between the received and replica signals and is an expression of the signal strength. SNR is defined as the ratio of the post-correlation signal power $P_{S}\;$to the noise power $P_{N}$ [36]:

(5) $$\mathrm{SNR}=10\cdot log\left(\frac{P_{S}}{P_{N}}\right)=10\cdot log{\left(\frac{S}{\sigma_{N}}\right)}^{2}\left[dB\right],$$

where S stands for amplitude of the correlation peak and $\sigma_{N}$ represents the standard deviation of the noise. SNR expresses the amount by which a signal level exceeds its noise in decibels. 

The carrier-to-noise ratio (C/N_0_) is identified as the carrier power divided by the noise power spectral density per unit bandwidth. It is expressed as follows [39]:

(6) $$C/N_{0}=C-\left(N-\mathrm{BW}\right)=C-N_{0}=\mathrm{SNR}-\mathrm{BW}.$$

where C represents the carrier power in dBm or dBW; N and $N_{0}$ represent the noise power and noise power density, respectively; and BW is the bandwidth of the observation. Typical values in an L1 C/A code receiver are: C/N_0_ ≈ 37–45 dB-Hz.

### 2.2. Localisation of a Moving Jammer

To characterise the signal and jammer power relationship, the following can be used: (a) the carrier-to-noise density power ratio C/N_0_, (b) the jammer-to-noise density power ratio J/N_0_, (c) the jammer-to-signal power ratio J/S, or (d) the jammer-to-noise power ratio J/N. An impact of GNSS jammers on consumer grade receivers is well established in Bono et al. [35], while this paper deals with an impact on surveyors’ professional receivers. For passing-by jammers, which emit intermediate power, a significant decrease in receiver performance may potentially cause even a professional GNSS receiver to lose its lock, which may at least extend the surveyors open field work. With information that such a disturbance from a steady velocity source is on the way, and from estimations of the distance or angle of arrival of the jammer-receiver, it is possible to estimate how long a degradation of functionality may last. The benefits of multi-constellation and multi-frequency receivers [35] for object positioning tracking in the presence of narrow band interfering sources are obvious.

The localisation of jammers is based on measurements of the effective carrier-to-noise density power ratio, C/N_0 *eff*_ [40], with an array of sensors at prior known locations. The approach of the moving interferer localisation is constrained by the relatively poor sensitivity of the averaged C/N_0_ of the satellites in view to the jammer-receiver distance *d* and also a relatively low sampling rate (1 s) due to the fast-moving jammer with velocities from 8.3 to 25.0 m/s. The relation can be modelled as, for example [35]:

(7) $$\frac{C_{avg}}{N_{0}}{|}_{eff}=\bar{\beta }+10\alpha \;\log_{10}\left(d\right).$$

However, the approach of the moving interferer localization, e.g., Betz [41], was enhanced by the known velocity of the jammer due to an in-lined located set of sensors. Assuming that at the lowest C/N_0_, the jammer was at the shortest distance to the receiver, the calibration of *α* and $\bar{\beta }$ is possible. Based on experimental data, these two parameters were established for each receiver for all drives enabling the localisation through bi-lateration with the pairs of synchronised receivers. 

### 2.3. Receiver Position and Dispersion of Coordinates While Jamming

A qualitative parameter used to measure the effect of jamming on the position determination should initially be defined. The distance between the real position and the measured one was a sensible choice. It is well known that the accuracy of GNSS receivers is much better in a horizontal rather than a vertical direction. For this reason, the projections of the distance to the horizontal plane and to the vertical line were calculated instead of the total distance itself.

The first step was to determine the real position from all the samples: northing ($n_{n}$), easting ($e_{n}$) and height ($v_{n}$). Since some of them were jammed, and therefore it was not a good idea to simply calculate the mean value of all of them; instead, the average was calculated using the weighted coefficients:

(8) $$\bar{a}=\frac{1}{N}\overset{N}{\underset{n=1}{\sum }}w_{n}a_{n},$$

where a is any of the quantities (n, e or v), $w_{n}$ the weighted function and *N* the number of samples. Similarly, the standard deviation could be obtained from:

(9) $$\sigma_{a}^{2}=\frac{1}{N-1}\overset{N}{\underset{n=1}{\sum }}w_{n}{\left(a_{n}-\bar{a}\right)}^{2}.$$

The weighting function should ideally be chosen such that it would be 1 for non-jammed samples and 0 otherwise. Although the jamming time was well known during the acquisition, an algorithm was used to determine the jamming presence in order to keep the procedure independent of the measurements. For this purpose, $w_{n}$ was defined iteratively. All $w_{n}$ were initially set to 1 in order to get a rough estimate of $\bar{a}$ and $\sigma_{a}$. In the next step, all samples that had at least one of the three quantities out of the $3\sigma_{a}$ region were considered jammed and $w_{n}$ was set to 0 for them. The new weighting function improved the accuracy of $\bar{a}$ and $\sigma_{a}$. The procedure was then repeated until no change in $w_{n}$ was observed. This typically occurred in five to six steps.

It should be stressed that the weighting function obtained in this way is not exact. There are some samples outside the $3\sigma_{a}$ region that are not jammed and there are some jammed samples within it. The question is how they affect the determination of $\bar{a}$ and $\sigma_{a}$. In order to get an estimate of the former, a normal distribution of the position is assumed for each of the three quantities when the signal is not jammed. This basically means that if there were no jammed samples, the procedure used for the purposes of this research would yield the solution s of the equation:

(10) $$\frac{1}{\sqrt{2\pi }\;\sigma }\overset{3s}{\underset{-3s}{\int }}x^{2}e^{-\frac{x^{2}}{2\sigma^{2}}}dx=us^{2}$$

instead of $\sigma_{a}$, where u is the normalisation factor (due to weighting):

(11) $$u=\frac{1}{\sqrt{2\pi }\;\sigma }\overset{3s}{\underset{-3s}{\int }}x^{2}e^{-\frac{x^{2}}{2\sigma^{2}}}dx.$$

The above equation can be easily solved numerically, resulting in the ratio s/σ=0.98306, meaning this effect can be simply ignored.

For the effect of jammed samples within the interval of interest to the increased σ estimation, the following assumptions will be made. The overall position distribution is composed of two normal distributions, each with its own deviation: $\sigma_{N}$ for non-jammed samples and $\sigma_{J}$ for jammed, where the former is under one centimetre while the latter is above one metre. Where r is the proportion of the jammed samples, the mean square of the weighted samples can be calculated using:

(12) $$us^{2}=\frac{1-r}{\sqrt{2\pi }\;\sigma_{N}}\overset{3s}{\underset{-3s}{\int }}x^{2}e^{-\frac{x^{2}}{2\sigma_{N}^{2}}}dx+\frac{r}{\sqrt{2\pi }\;\sigma_{J}}\overset{3s}{\underset{-3s}{\int }}x^{2}e^{-\frac{x^{2}}{2\sigma_{J}^{2}}}dx,$$

with u again being the normalisation factor. Since s should obviously be greater than 0.98306σ≈ σ and 99.7% of non-jammed samples fall within an 3σ interval, the first integral can be extended to infinity. This reduces the first term to $\left(1-r\right)\sigma_{N}^{2}$. Meanwhile, $\sigma_{N}$ (and consequently s) is less than $\sigma_{J}$ for at least a factor of 100. This means that the exponential function in the second term can be approximated to 1 within the interval of integration with the error of order $x^{2}/\sigma_{N}^{2}$, which can easily be neglected. A similar reasoning can be applied to the calculation of u. With these simplifications, the above formula can be reduced to:

(13) $$s^{2}=\frac{\left(1-r\right)\sigma_{N}^{2}+2\frac{r}{\sqrt{2\pi }\;\sigma_{J}}\frac{{\left(3s\right)}^{3}}{3}}{1-r+\frac{r}{\sqrt{2\pi }\;\sigma_{J}}6s}.$$

Although the above formula could be solved numerically for a particular r and the ratio $\sigma_{N}/\sigma_{J}$, it is useful to get an analytical approximation for the solution. Since $s\approx \sigma_{N}$, a new variable h will be introduced such that $s=\left(1+h\right)\sigma_{N}$. Assuming both h and $\sigma_{N}/\sigma_{J}$ are small and linearising the above expression in both terms (that includes neglecting all the terms $h\sigma_{N}$), an expression for the approximate solution of the above equation can be found:

(14) $$h=\frac{6}{\sqrt{2\pi }}\frac{r}{1-r}\frac{\sigma_{N}}{\sigma_{J}}.$$

Since $\sigma_{J}$ is at least 100 times greater than $\sigma_{N}$, the resulting *s* varies from $\sigma_{N}$ by an order of 1%, which can again be neglected.

The above reasoning gives a good justification for using such a method for the mean value and standard deviation calculation. Once the mean values were obtained, the position discrepancies were defined as:

(15) $$\delta H_{n}=\sqrt{{\left(n_{n}-\bar{n}\right)}^{2}+{\left(e_{n}-\bar{e}\right)}^{2}}$$

and:

(16) $$\delta V_{n}=\left|v_{n}-\bar{v}\right|$$

for the horizontal and vertical error respectively.

## 3. Setup and Measurement Campaign

**Figure 1:**
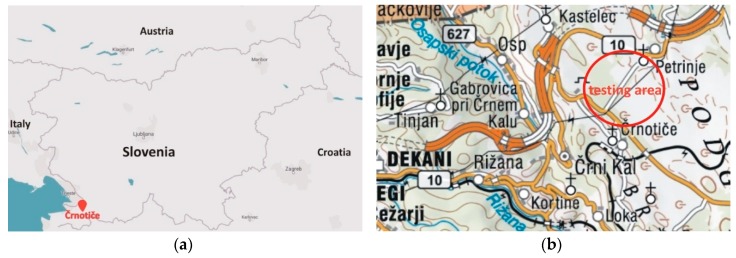
Geographic position of the testing area: (**a**) location of the village Črnotiče in Slovenia and (**b**) approximate location of the testing area (red circle) [42,43].

The testing area was established in July 2015 close to the village of Črnotiče in Slovenia (Figure 1). The main reasons that this location was chosen was that it is a remote area with a minimal impact of jamming for users and because several different jamming experiments have already been successfully conducted there. Moreover, there are no elevated obstacles near the location of the GNSS receivers that would disturb the GNSS signal reception, there is almost no traffic, and the straight road enables the vehicle to be run at a constant speed. The most important fact is that the use of a jammer was authorised by the Agency for Communication Networks and Services of the Republic of Slovenia (AKOS). In July 2019, open field GNSS measurements were performed in order to carry out experiments of the vulnerability of different GNSS instruments under real-life jamming conditions.

### 3.1. GNSS Geodetic Receivers Used in the Experiment

**Figure 2:**
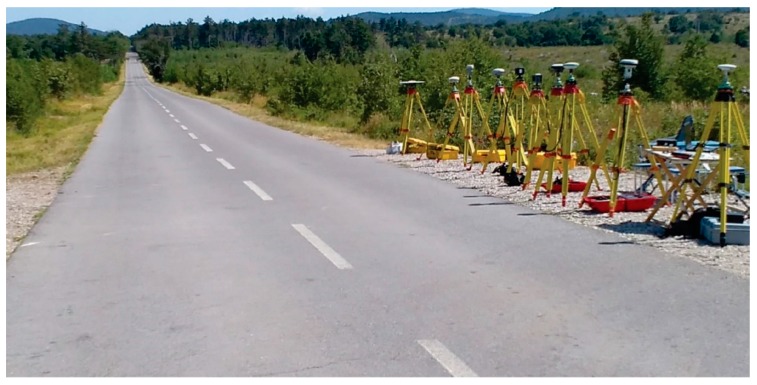
The instrument set up at point *B* from north-east (left) to south-west (right) at the location (own study).

Several different GNSS-receivers from Trimble Inc. (Sunnyvale, CA, USA), Javad GNSS Inc. (San Jose, CA, USA), Leica Geosystems AG (Heerbrugg, Switzerland) with specific antenna types were used in the experiments, namely: Trimble 4000 SSi (antenna type: TRM22020.00+GP), Trimble R8 (antenna type: TRMR8_GNSS), Trimble R10 (antenna type: TRMR10), Trimble R8s (antenna type: TRMR8S), Javad Triumph-VS (antenna type: JAVTRIUMPH_VS), Javad Triumph-LSA (antenna type: JAVTRIUMPH_LSA), Leica GS18 (antenna type: LEIGS18), Leica GS07 (antenna type: LEIGS07) and Leica GS15 (antenna type: LEIGS15) (see Figure 2). Some were only able to receive GPS signals (the oldest receiver, Trimble 4000 SSi), most of them received GPS and GLONASS signals, while some were also able to receive other signals, for example, from Galileo (Javad Triumph-VS, Javad Triumph-LSA, Trimble R10, Leica GS15 and Leica GS18). As can be seen from Figure 2, the instruments were set on tripods and tribrach (positions are given in Table 1) close to each other.

### 3.2. GNSS Jammer Characterisation

**Figure 3:**
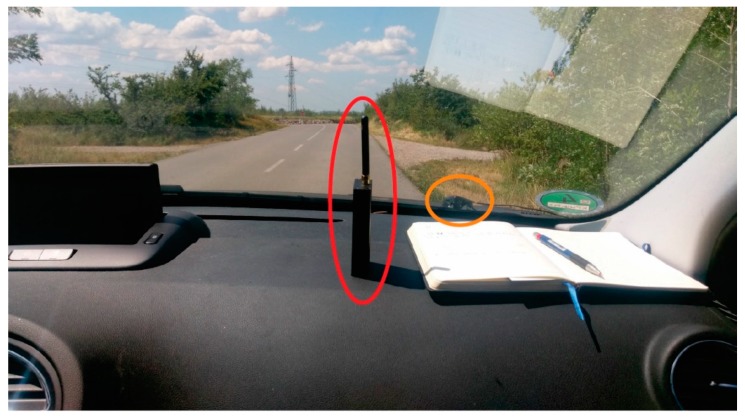
Position of the battery jammer (red ellipse) on the front panel and the GNSS receiver’s (Ublox, NEO 6T) patch antenna on outer windscreen surface (orange ellipse) (own study).

For the benefit of the experiment, a commercially available in-car jammer was used. With regard to Borio et al. [35], it was a sub-miniature version A (SMA) unlabelled jammer L1/E1 jammer, with no manufacturer’s data, powered by a battery, its external antenna with omnidirectional radiation pattern was connected through a SMA connector (see Figure 3), which emitted a single saw-tooth chirp signal, according to References [44,45,46], belonging to the class II group. According to their tests, the jammer’s output features a period of 10 μs, while according to our test it, the device raises noise power up to 50 dB in a frequency band of 1570 MHz ± 20 MHz. Following the STRIKE3 (Standardisation of GNSS Threat reporting and Receiver testing through International Knowledge Exchange, Experimentation and Exploitation) attempt of standardised threat reporting of jamming events [15], the effect of this particular jammer should be described as an interference event Type B, exerting a 10 dB decrease of C/N_0_ lasting for more than 5 s.

## 4. Experiments

**Figure 4:**
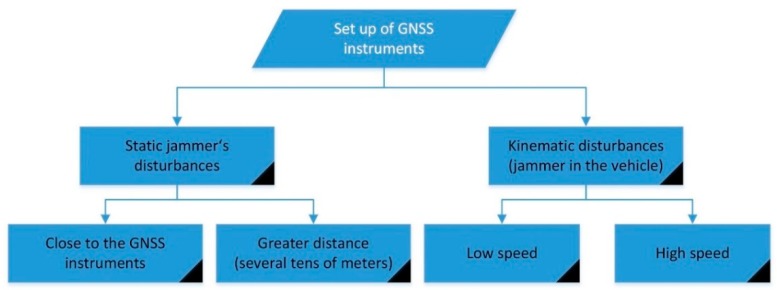
Pre-planned measurement scenarios (own study).

**Figure 5:**
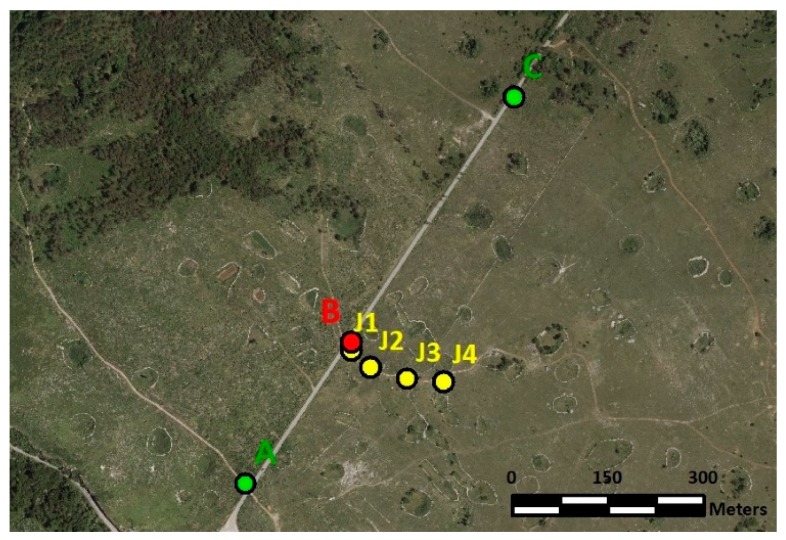
Location of the most distant two-way points (*A* and *C*) for kinematic experiments (green), location of the receivers (*B*) in red and static positions of the jammer (J1, J2, J3 and J4) in yellow (own study).

The experiments followed a pre-planned library of scenarios for jamming detection (see Figure 4). The jammer was initially kept static at different distances from the GNSS receivers (approximately 10 m, 50 m, 100 m and 150 m) at positions indicated by J1–J4 (Figure 5, Table 2). Kinematic jamming was then performed using a jammer located in a vehicle, which moved back and forth between points *A* and *C* by passing the GNSS instruments at location *B* (see Table 1). In each static jammer’s occupation, two 3-minute jammings were performed, where the jammer was first placed vertically and then in a horizontal position. Several minutes of non-interruption were conducted between each successive session.

Experiments involving jamming with the static position of the jammer at the specific point were conducted first; namely, two 3-minute jammings were performed at each of the positions indicated by J1, J2, J3 and J4 (Table 3), which incorporated approximate jammer-receiver distances and the start/end times of the static jamming.

**Figure 6:**
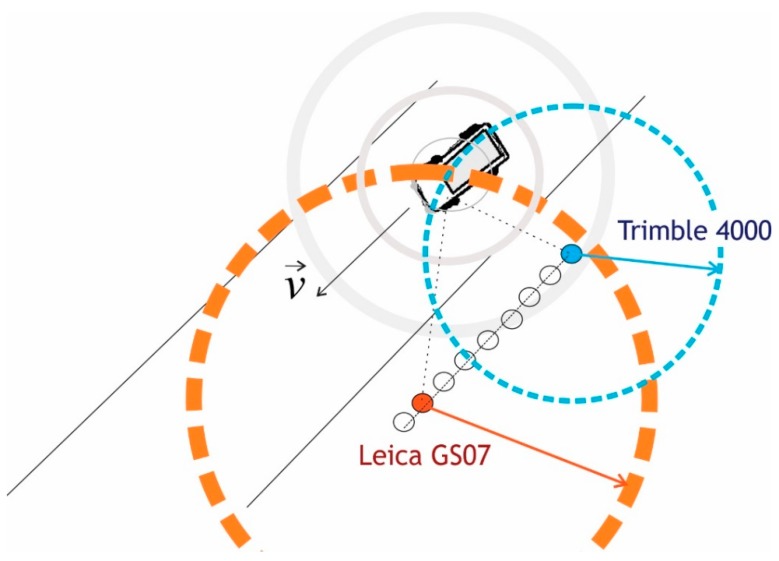
The principle of localising of the moving jammer in a vehicle by an array of GNSS receivers (own study).

Additionally, kinematic tests were performed (Figure 6), whereby a jammer was placed in a vehicle that moved back and forth between points *A* and *C* and passed the receivers’ location at different speeds several times, namely 30 km/h, 60 km/h and 90 km/h. Several tests were performed at the specific speed of the vehicle. The maximum distances between the jammer and the GNSS instruments were about 400 m (between *A* and *B*, as well as between *B* and *C*). The speed of the vehicle and the moment the vehicle passed the instruments are shown in Table 4. Jammer localisation relies on lateration using the best estimations of the distance from the pairs of synchronized geolocated GNSS receivers, as presented on Figure 6.

## 5. Data Analysis, Results and Discussion

The processing of GNSS observations was performed in version 2.3.2 of Leica Infinity (Leica Geosystems AG, Heerbrugg, Switzerland) and version 2.4.3, b33 of RTKlib [47]. In the field, the coordinates were also acquired in RTK mode for most receivers (not for Trimble 4000 SSi), and for detail, post-study static observations at a time rate of one second were collected. Post-processing generally results in a more accurate, comprehensive solution than is possible in real-time and offers a great deal of flexibility, especially since the applications can involve stationary or moving processing strategies, as well as quality checks of the observations. In the processing, the “continuous method” of ambiguity integer resolutions allowed for integer ambiguities to be estimated in every single epoch.

**Figure 7:**
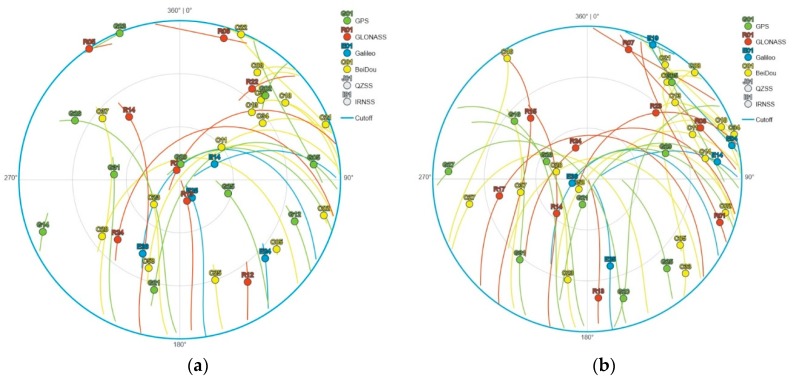
Skyplot of visible satellites for 16 July 2019: (**a**) at the beginning of the experiment 08:20:00 UTC and (**b**) at the end of the experiment 09:58:00 UTC [48].

The input data for this research were RINEX-files from 1-second static measurements from 08:20:00 to 10:00:00 UTC. Figure 7 represents the satellite constellation at the start and end time, while the timespans, which show the availability of each specific satellite from all nine tested receivers, are shown in the Appendix A. Reference coordinates for each of the receivers were gained from static relative processing. A virtual reference station (VRS), which was generated from the Slovenian CORS network SIGNAL (Slovenia (SI)-Geodesy-NAvigation-Location), was used as a base station. Since the authors wanted to show the effect of jammers at each of the specific registrations, the kinematic positions were acquired using post-processing. 

From quality checks, the C/N_0_ values for all satellites in view for each of the receivers were obtained in order to study each jammer’s location.

### 5.1. Results of the Jammer Detection and Its Localisation

**Figure 8:**
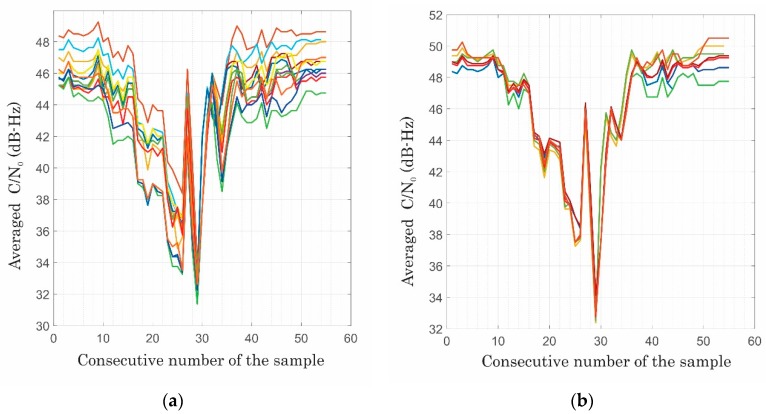
Variations of an average of C/N_0_ on a Leica GS15 due to the passage of the jammer and its sensitivity to satellite selection within hemispheres of varying azimuths: (**a**) elevations above 10° and (**b**) above 40° (own study).

In order to obtain the best jammer positioning lateration performance of down to 10 m for a jammer closer than 50 m approaching at 30 km/h (a decrease of the C/N_0_ in Figure 8), the proportionality parameter *α* (consider Equation (7)) was typically 1.0, while $\bar{\beta }\;$was around 30 for the surveying receivers. For the consumer grade (Ublox, M8N), *α* was typically over 1.0, whereas $\bar{\beta }\;$was less than 20.

**Figure 9:**
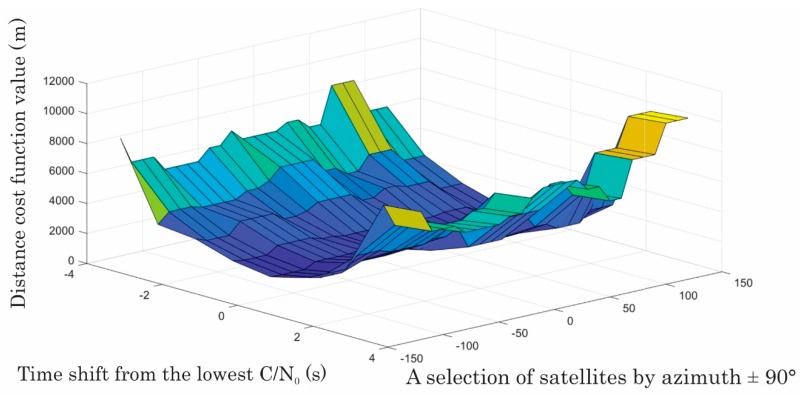
Sensitivity of the distance related cost function on satellite selection by azimuth at a minimum elevation of 35°, and the time shift of C/N_0_ vs. the closest distance jammer-receiver for Leica GS15 during the last test at 90 km/h (own study).

Forming a distance/cost function for each drive from the sum of the differences of true distances and the best estimated distance gave an insight into the influence of selection of the satellites’ signals that were used for averaging C/N_0_, both for azimuth and elevation. The sensitivity to the selection of satellites can be seen in Figure 9. Moreover, the influence of shifting the minimum of the C/N_0_ pattern with respect to the moment of the closest point of the receiver and jammer is also given (see Figure 9). Interestingly, applying the non-jammed L2-frequency observations (1227.60 MHz) C/N_0_ generally did not give significantly worse results than on L1, since the effect on C/N_0_ within L2 occurs due to the processing reasons. 

However, jammer localisation is only of side importance for the surveyor, with estimated distances, followed by laterations from pairs of receivers. A surveyor may assume from the disturbance that it may decay due to its moving in or it may last until the jammer is turned off. 

### 5.2. Positioning Results

The results from both the static and kinematic jamming tests showed that the jammer affected the measurements of the specific receivers differently. Furthermore, GLONASS signals from some receivers were more resistant to jamming. This can likely be attributed to the higher operating frequencies and different frequencies of GLONASS satellites.

**Figure A1:**
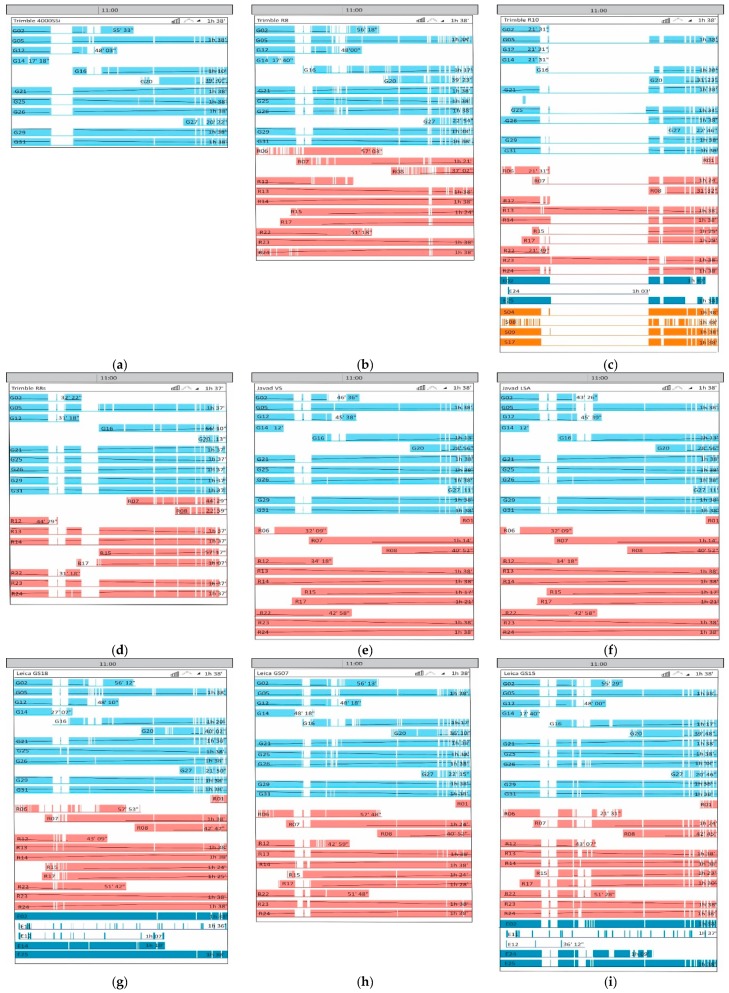
Timespans of observations for satellites in view (cyan for GPS (annotation G), red for GLONASS (annotation R), blue for Galileo (annotation E), orange for SBAS (annotation S) acquired from Lieca Infinity 2.3.2 for the receivers: (**a**) Trimble 4000 SSi, (**b**) Trimble R8, (**c**) Trimble R10, (**d**) Trimble R8s, (**e**) Javad Triumph-VS, (**f**) Javad Triumph-LSA, (**g**) Leica GS18, (**h**) Leica GS07, and (**i**) Leica GS15. Times refer to local time, which is UTC+2h. The black lines in each particular satellite timespan show the elevation angles of the satellite.

The oldest receiver used in this study, Trimble 4000 SSi, was a GPS-only receiver, manufactured in the mid-1990s. The authors deliberately investigated its performance to investigate the benefits of the new receivers. While jamming in the vicinity of the receiver at a distance of about 10 metres twice in succession, unlike other instruments, it did not detect the end of the disruption so quickly, thus there were missing observations for a period of six minutes (see Figure A1a). Based on its age, it performed surprisingly well in the other jamming scenarios.

When comparing newer instruments, it could be said that the L1/E1 jammer also significantly influenced the acceptance of GLONASS signals, especially in the case of Trimble R8, Leica GS07 and Leica GS15. Meanwhile, Trimble R8, both of Javad’s receivers and the latest Leica GS18 instrument performed much better regarding GLONASS reception (see Appendix A).

**Figure 10:**
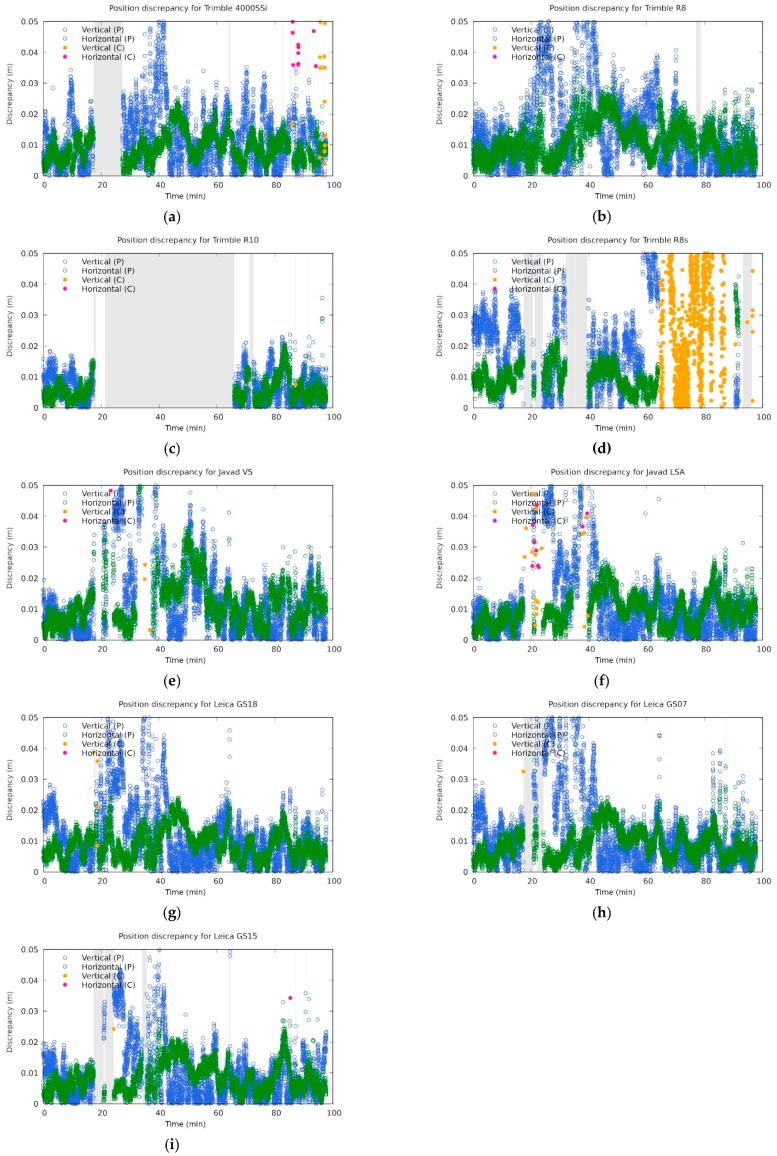
Position discrepancies for the involved instruments: (**a**) Trimble 4000 SSi, (**b**) Trimble R8, (**c**) Trimble R10, (**d**) Trimble R8s, (**e**) Javad Triumph-VS, (**f**) Javad Triumph-LSA, (**g**) Leica GS18, (**h**) Leica GS07, and (**i**) Leica GS15 in the region up to 5 cm. Annotations P and C mean phase fix and code solution respectively. The grey regions are the intervals where the instruments did not report any position. The time origin was set to 08:20 UTC time. Code solutions mostly fall outside of the plotting region and are thus not shown.

The performance of the instruments was then evaluated according to their ability to calculate their position and the discrepancy of the solution from the exact value (Figure 10). The grey region shows the time interval where an instrument was unable to give any kind of solution. The green and blue circles represent the horizontal and vertical discrepancy in the case when a phase solution was given. The magenta and yellow circles represent those events when an instrument was able to get a code solution only. It should be stressed that most of the code solutions (and some phase solutions) fell outside the plotting region (up to 5 cm), because their error was typically far beyond that value (the region of interest for geodesy).

**Figure 11:**
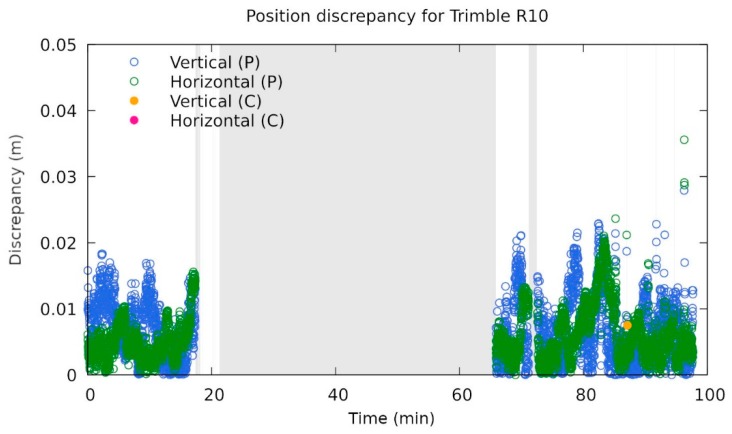
Vertical and horizontal errors during jamming for the Trimble R10 receiver. The time origin was set at 08:20 UTC time. The annotations P and C indicate the type of solution used, namely phase and code, respectively. The grey region represents the interval when no position was given from the receiver (own study).

It seems that the receiver most affected in the static jamming scenario was Trimble R10, which did not provide its position for more than 40 min, although the jamming took significantly less time (see Figure 10c, in detail in Figure 11). The positioning failed immediately after the first static jamming in the vicinity of the receiver (point J1 at Figure 5) and performed well when final static jamming was performed again at point J1. This is not necessarily a bad property, since for end users it is better to get no results than to get bad ones.

The other receivers had significantly shorter blackouts (the grey region in Figure 10 and Figure 11). In particular, the two Javad’s instruments were able to give some solution (at least a code one) throughout the jamming campaign. It should be also stressed that most of the code solutions and some carrier-phase solutions are not seen in those graphs from Figure 10 due to the plotting scale.

**Figure 12:**
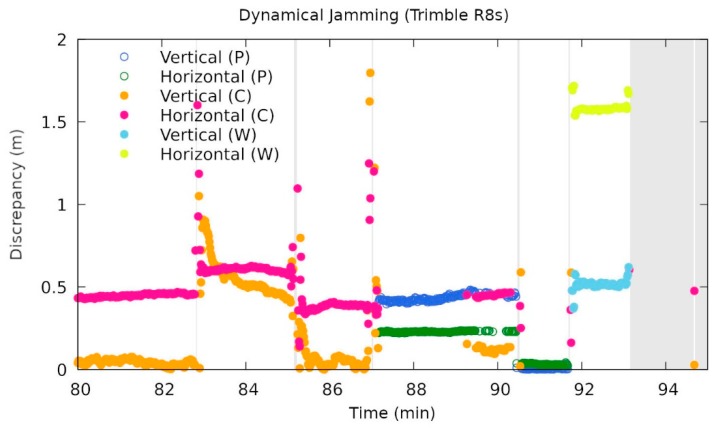
A zoomed area of the vertical and horizontal discrepancies during the dynamic jamming for the Trimble R8s receiver. W stands for the widelane ambiguity calculation. Note the wrong fixing in the interval from 87 to 90 min from the beginning of the experiment (08:20 UTC) (own study).

The latter indeed happened to another Trimble instrument, namely R8s (see Figure 12), around 09:47 UTC (87 min from 08:20). At the beginning, it was unable to get a phase fix and reported the code solution instead. Then suddenly (at 09:47), it jumped to a wrong phase fix and stayed there for approximately two minutes. Thereafter, it alternated between a wrong phase fix and the code solution for a minute. In the next minute it reported the correct phase solution, then finally reported the widelane solution, and after a minute, stopped reporting.

Since this is the only such event during this jamming campaign, it is not necessary the case that Trimble R8s is the only instrument that can be affected in such a manner. Furthermore, it is not clear whether this event was isolated or if it should be considered a deficiency of the instrument itself. The authors intend to carry out some further research in this area in the near future.

**Figure 13:**
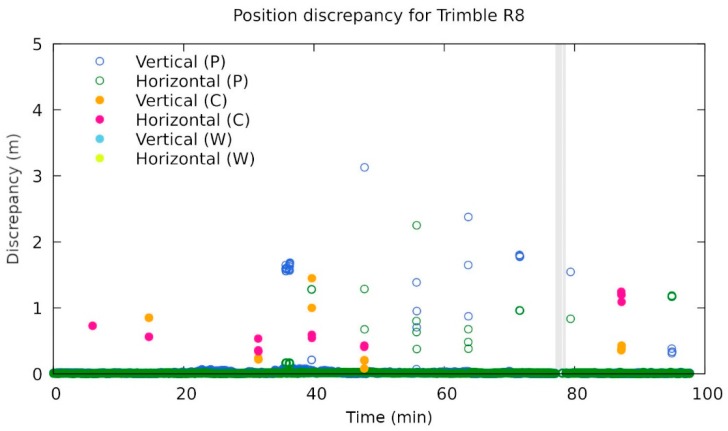
Discrepancies of the reported position for Trimble R8 (note the zoomed scale). See Figure 12 for a detailed description of the annotations. Those points that are above 1 m and reported as a phase fix were particularly problematic (own study).

Another Trimble instrument that did not handle the jamming well was Trimble R8. There were some points that were reported as a phase fix but differed by a few metres from the exact position (see Figure 13). However, those points were isolated in the sense that the wrong fix lasted for a single acquisition only and returning to within normal values afterwards.

**Figure 14:**
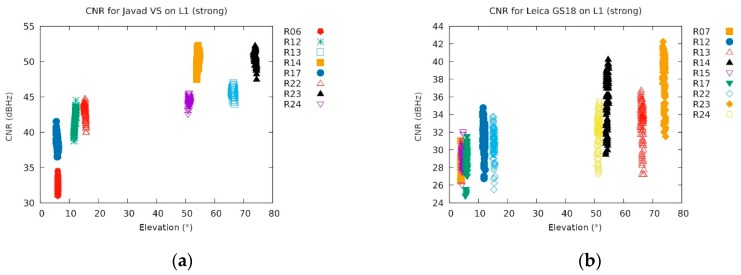
Carrier-to-noise ratio (C/N_0_; denoted: CNR) for (**a**) Javad Triumph-VS and (**b**) Leica GS18 during strong jamming (09:38 till 09:40). Note that only GLONASS satellites were received in the meantime (the assignation with the letter R). The elevation was also plotted in order to get rid of its effects. The lowest levels of R24 could be attributed to malfunctioning since the satellite shut down shortly after (own study).

Returning back to the discussion of GLONASS reception, it seems that the two Javad instruments, namely Javad Triumph-LSA and Javad Triumph-VS, were best able to manage data from the GLONASS satellites. The carrier-to-noise ratio (C/N_0_) for Javad Triumph-VS and Leica GS18 can be seen in Figure 14. The former had stronger signals of about 10 dBHz and did not manifest such big drops. Although none of the instruments were able to get a phase fix from those, they were able to keep a code solution that enabled them to recover to the phase fix faster after the jamming ended.

In principle, the following instruments were able to receive the Galileo signal: Trimble R10, Leica GS15, Leica GS18, Javad Triumph-LSA and Javad Triumph-VS (see Appendix A). The Javad receivers obviously cancelled the Galileo signal reception due to a failure of Galileo. As a result of the problem with the operation of Trimble R10, which also provided BeiDou signals, it was not possible to provide a final analysis for this navigation system. It can be seen that the received Galileo signals, interestingly, were not as disturbed as those of the GPS, although they shared the same frequencies. When comparing Leica GS15 and Leica GS18 in terms of Galileo signals, a significant improvement could be seen in the operation of the newer Leica GS18. This finding will be the focus of further research in which the authors will include a larger number of instruments from many other manufacturers.

**Figure 15:**
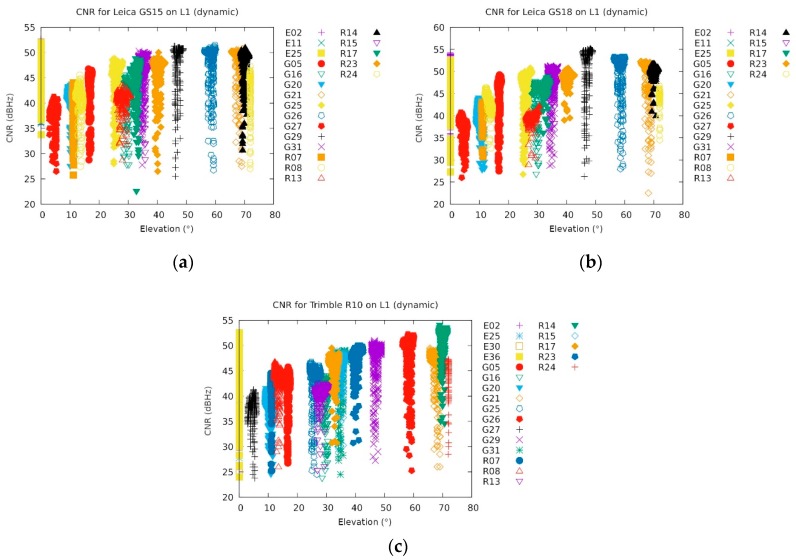
Carrier-to-noise ratio during the dynamic jamming for the Galileo ready receivers: (**a**) Leica GS15, (**b**) Leica GS18 and (**c**) Trimble R10 (the two Javad’s instruments are not shown since they ignored Galileo because it was not operational). All the Galileo satellites (annotation E in front of the number) reported an elevation of 0°, which can be attributed to malfunctioning of the Galileo system during the jamming campaign (own study).

However, due to malfunctioning of the Galileo system during the jamming campaign, the signal was useless for the position determination. The two Javads completely ignored Galileo, while the other three gave an elevation of 0°, probably due to the invalid ephemeris information (see Figure 15). However, the information could be retrieved only when the jamming was not too strong. Galileo signals are transmitted in the same frequency band as GPS. Therefore, they are expected to be affected by the jammer in a similar way.

Table 5 summarises a review of the effective positioning of both the code and carrier phase, the latter using ambiguity fixed values, for each of the receivers from 08:20 to 10:00 UTC, while static and kinematic jamming were performed. Furthermore, the quality of positioning is given within the accuracy of RTK positioning. 

Table 6 summarises maximal deviations in the code- and carrier-phase-based positioning, obtained from 08:20 to 10:00 UTC, during static and kinematic jamming. It is obvious that Trimble R8s and Trimble 4000 SSi reported completely wrong code-based positions for some moments. Moreover, Trimble R8 was giving a false carrier-phase solution for some time, which differed by a few metres from the exact solution and which should be considered a concern for geodetic use. Interestingly, small errors in carrier-phase positioning existed for the outdated instrument Trimble 4000 SSi, which is based only on GPS.

As mentioned before, Trimble R10 had problems in positioning, which failed immediately after the first jamming in the vicinity of the receiver. Obviously, the instrument recognised some malicious events and stopped reporting positions, which could be treated as beneficial. Unfortunately, it did not recognise the end of the jamming. However, other code and carrier-phase solutions, acquired during short kinematic jamming events, were within the expected positioning accuracy. Positioning results for other receivers were within the expected range of the accuracy.

## 6. Conclusions

Experiments investigating the impact of low cost L1/E1 jammers on the functioning of several GNSS instruments are complex, largely because they cannot be performed anywhere and at any time. For the purposes of this research, GNSS observations that were acquired from several geodetic GNSS receivers placed at the very same location were simultaneously processed in order to get information about the vulnerability of each specific GNSS geodetic receiver with the knowledge of the relevant authorities.

Although the observation plan was prepared with the utmost care, the realisation cannot always be optimal. Legal testing of jamming at the specific time was assured; however, the time of the measurements unfortunately coincided with technical problems with Galileo that occurred in July 2019 due to an outage in the ephemeris provisioning [49]. Therefore, since not all the receivers that were at our disposal for this research could receive Galileo signals, the study was limited to just GPS and GLONASS. Galileo signals were nevertheless received (see Appendix A); however, further computations of the elevations and azimuth due to the lack of the ephemerides presented a problem for the analysis.

That being said, an analysis of the effect of jammers on the geodetic positioning instruments was carried out. It has been shown that all the instruments should be able in principle to detect the presence of a jammer, and to some extent, even localise its position. Applying the distance evaluation procedure, we get the most accurate solutions using the L2 band C/N_0_ data from Trimble 4000 SSi. Leica GS07 results are more significant in bilateration according to the Leica GS15 receiver. It was also shown that the jammer significantly deteriorated the performance of the receivers by means of position determination. When strong jamming was taking place (jammer in the receiver’s vicinity), virtually all the instruments were unable to obtain a fix (a phase solution) at least for some time. However, both the Javads and Leica GS18 performed slightly better, providing at least a code solution all the time. On the other hand, Trimble R10, and to some extent Trimble R8s, had a significant suppression time where they provided no solution at all. Furthermore, Trimble R8s was giving a false phase solution for some time, which should be considered a concern for geodetic use. At the end, the effect on satellite’s CNR has been calculated, showing that both Javads performed better by means of GLONASS reception. In the other instruments, the GLONASS reception was affected by the jammer’s presence despite that particular jammer not interfering significantly with the frequency band used by GLONASS satellites.

Performing experiments of this kind and interpreting the results is always limited in some ways. There are many factors in the complete workflow that influence the results. Nevertheless, the authors believe that the results and conclusions from their experiments contribute to a better understanding of the impact of issues in everyday use of GNSS in geodetic applications, as well as in civil use for navigation. To this end, further work will be focused on identifying the causes of false ambiguity fixing and the quality of jammer’s localisation in several different jamming scenarios.

## Figures and Tables

**Table 1:** Locations of instruments at location *B* in the Slovenian realisation of the coordinate system ETRS89; instruments were set up in a line. Heights H were acquired from the ellipsoidal heights h by using the SLO_VRP2016/Koper geoid model.

Receiver Type	B-Latitude	L-Longitude	h (m)	H (m)
Trimble 4000 SSi	45°33′49.44330″ N	13°53′38.49922″ E	480.435	435.118
Trimble R8	45°33′49.38705″ N	13°53′38.46655″ E	480.509	435.192
Trimble R10	45°33′49.34812″ N	13°53′38.42442″ E	480.833	435.516
Trimble R8s	45°33′49.30974″ N	13°53′38.39439″ E	480.662	435.345
Javad Triumph-VS	45°33′49.27689″ N	13°53′38.36147″ E	480.676	435.359
Javad Triumph-LSA	45°33′49.24172″ N	13°53′38.31830″ E	480.589	435.272
Leica GS18	45°33′49.21000″ N	13°53′38.28117″ E	480.742	435.425
Leica GS07	45°33′49.18701″ N	13°53′38.24986″ E	480.782	435.466
Leica GS15	45°33′49.14962″ N	13°53′38.22753″ E	480.701	435.384

**Table 2:** Position of the jammer in static mode (3-minute jammings, where the jammer remained fixed in a certain position). Heights H were acquired from the ellipsoidal heights by using the SLO_VRP2016/Koper geoid model.

Point Name	B-Latitude	L-Longitude	H [m]
J1	45°33′49.06956″ N	13°53′38.52143″ E	435.484
J2	45°33′48.18814″ N	13°53′39.88645″ E	435.214
J3	45°33′47.62954″ N	13°53′42.58632″ E	433.616
J4	45°33′47.53317″ N	13°53′45.21659″ E	433.911

**Table 3:** Static positions of the jammers and times of the 3-minute jammings.

Distance of Jammer Instruments	Point of the Jammer’s Location	Polarisation of the Jammer	Start and End Times of Jamming (UTC)
12 m	J1	Vertical	08:37:21–08:40:37
		Horizontal	08:41:11–08:44:10
50 m	J2	Vertical	08:53:51–08:56:54
		Horizontal	08:57:25–09:00:19
100 m	J3	Vertical	09:03:03–09:06:16
		Horizontal	09:07:28–09:10:34
160 m	J4	Vertical	09:13:44–09:17:36
		Horizontal	09:18:09–09:20:58
12 m	J1	Vertical	09:24:01–09:24:23

**Table 4:** Jammer’s kinematic disturbances (jammer in the vehicle).

Speed of the Vehicle	Time at Which the Vehicle Passed the Instruments (UTC)
30 km/h	09:42:48
30 km/h	09:45:11
30 km/h	09:46:58
60 km/h	09:50:28
60 km/h	09:51:40
80 km/h	09:53:09
90 km/h	09:54:40
90 km/h	09:56:17

**Table 5:** Percentage of the successful ambiguity fixing from 8:20 to 10:00 UTC, i.e., during static and kinematic jammings.

Receiver Type	Any Solution	Phase Fix	Horizontal < 1 cm	Vertical < 2 cm
Trimble 4000 SSi	88%	83%	51%	68%
Trimble R8	99%	98%	45%	73%
Trimble R10	52%	49%	42%	48%
Trimble R8s	81%	55%	29%	33%
Javad Triumph-VS	100%	89%	41%	71%
Javad Triumph-LSA	100%	91%	50%	77%
Leica GS18	99%	99%	61%	82%
Leica GS07	94%	94%	57%	76%
Leica GS15	91%	89%	59%	79%

**Table 6:** Quality of positioning: maximal horizontal and vertical deviations in coordinates in the horizontal plane and in heights during static and kinematic jammings.

	Maximal Error from Code Solutions (m)		Maximal Error from Phase Solutions (m)	
Receiver Type	Horizontal	Vertical	Horizontal	Vertical
Trimble 4000 SSi	143.67	426.69	0.025	0.060
Trimble R8	1.24	1.45	2.251	3.128
Trimble R10	2.91	4.38	0.036	0.085
Trimble R8s	772.01	1426.60	0.237	0.483
Javad Triumph-VS	2.92	7.16	0.096	0.127
Javad Triumph-LSA	1.71	3.72	0.092	0.496
Leica GS18	2.45	2.10	0.057	0.077
Leica GS07	2.31	2.50	0.066	0.163
Leica GS15	4.29	4.52	0.070	0.095
